# Exploring Well-Being and Its Correlates among Young Men Using Sen’s Capability Approach: Results from the Young Adults Survey, Switzerland (YASS)

**DOI:** 10.3390/ijerph19031247

**Published:** 2022-01-22

**Authors:** Susanne Ferschl, Peter Gelius, Karim Abu-Omar, Maike Till, Richard Benkert, Thomas Abel

**Affiliations:** 1 Department of Sport and Health Sciences, Technical University of Munich, 80992 Munich, Germany; 2 Department of Sport Science and Sport, University of Erlangen-Nuremberg, 91058 Erlangen, Germany; peter.gelius@fau.de (P.G.); karim.abu-omar@fau.de (K.A.-O.); maike.till@fau.de (M.T.); 3 Institute of Social and Preventive Medicine, University of Bern, 3012 Bern, Switzerland; richard.benkert@ispm.unibe.ch (R.B.); thomas.abel@ispm.unibe.ch (T.A.)

**Keywords:** young adulthood, well-being, capability approach, social inequalities

## Abstract

Background: The range of options people have to do the things they value in life may have strong effects on their well-being. This is especially true for young adults, as their opportunities and choices may affect both their current and future lives. This study follows Sen’s capability approach (CA) to assess young people’s well-being in terms of capabilities and functionings. Methods: Repeated cross-sectional data from the Young Adult Survey Switzerland were used for the analysis (N = 58,615). Descriptive statistics were applied to analyze the distribution of capabilities and functionings according to individuals’ capital equipment. Finally, multivariate regression analyses were conducted to investigate associations between social, cultural, and economic capital; overall capabilities; and functionings. Results: Young men with lower capital equipment rated their capabilities and functionings lower than others. Capabilities and corresponding functionings differed in the domains of health, happiness, and intellectual stimulation. Multivariate analysis confirmed the effects of social, economic, and cultural capital on both overall capabilities and functionings. Conclusions: Young men differ in their perceived capabilities and functionings in different life domains according to their equipment with different forms of capital. To better understand the mechanisms underlying the transformation of capabilities into functionings, future studies should analyze issues of choice and adaptation to restricted living conditions.

## 1. Introduction

Emerging adulthood, “a substantial number of years between the time young people reach the end of adolescence (around age 18) and the time they enter stable adult roles in love and work […]” [1] (p. 8), offers unique opportunities to explore different possible future paths of life [1,2]. It has been shown that young adults’ opportunities influence their choices in different life domains as well as their current and later well-being [3,4,5]. As such, young adulthood represents a critical phase in life. The purpose of this study was to explore young men’s well-being and its social correlates among the population group of young men in Switzerland. In doing so, this study follows the call to treat young adults as a distinct sub-population in research and policy [3], as well as to investigate the influence of socioeconomic factors on health specifically among the little researched group of emerging adult men [6]. 

In attempting to assess well-being, one has to recognize that this concept is complex and multi-dimensional [7]. At minimum, it means to judge life positively and to feel good [8]. In its broadest sense, it encompasses social, physical, and mental domains [8]. There is evidence that young adult women and men differ in their health and well-being profiles [9,10,11], which are influenced by socio-cultural factors such as gender-specific vulnerabilities and gender norms [9,12]. For instance, longitudinal research [13] has shown that a higher socio-economic background and higher social support both predict young people’s mental well-being. However, these factors showed different interaction mechanisms between men and women [13]. While gender equity has been recognized as an important determinant of health and well-being, knowledge and action on this topic among young adult men is still less common than among young women [12,14]. In particular, further research on the influence of socioeconomic factors on health and well-being among the male population is needed [6] in order to gain a robust evidence base informing suitable recommendations for prevention and intervention, taking into account the their unique well-being needs [15]. 

In attempting to investigate well-being, the CA provides a helpful framework to better understand how opportunities affect well-being. Its advantage lies in its multi-dimensional evaluative space including seven life domains. As existing well-being studies among young men have mostly investigated mental or psychological [10,11,13] or social well-being [16], the application of the CA might provide a more extensive well-being picture. Moreover, the focus on capabilities and functionings permits the assessment of well-being beyond the provision of resources or utility-based considerations [17,18]. Nevertheless, the CA does account for socio-economic conditions and resources [19,20], which are additionally regarded through the lens of Bourdieu’s theory of capitals in this study.

### 1.1. Sen’s Capability Approach

The CA views well-being as “the freedom of choice to achieve the things in life which one has reason to value most” [21] (p. 1161). The CA suggests focusing on what people can do and can be rather than solely on what they have [22] and differentiates between capabilities and functionings. Functionings (or well-being achievements) refer to the actual life conditions of people, e.g., the level of health, income, or nourishment a person can achieve [23]. Capabilities (or well-being freedoms) refer to “the alternative combinations of things a person is able to do or to be—the various ‘functionings’ he or she can achieve” [23] (p. 30) and reflect a person’s freedom to choose among a set of alternative functionings that they value most [24]. The choices an individual makes depend on the available capabilities, which in turn impact well-being outcomes [21]. Additionally, available resources as well as individual, social, and environmental characteristics enhance or constrain an individual’s choices and therefore well-being outcomes [21,25]. Due to the element of choice but also due to differing individual ideas about well-being, the CA suggests that capabilities theoretically are larger than their corresponding functionings. Consequently, capabilities and related functionings might be correlated with each other, albeit not in a perfect way. With the relevance of equally distributed capabilities being one of the CA’s core facets [26], the framework has also been suggested as a suitable approach to analyze social disparities across groups [27]. Regarding the material and non-material resources people have at their disposal to create capabilities [28], Bourdieu’s [29] theory of capital interactions has proved to be a helpful complementary theory [30,31,32,33]. According to Bourdieu [29], economic capital refers to money or other forms of material assets such as income or property; cultural capital was described to appear in three forms, namely, as incorporated cultural capital (skills and knowledge), institutionalized cultural capital (educational degrees and certificates), and objectivized cultural capital (books and tools) [29]; and social capital, finally, refers to different kinds of resources that can be mobilized through social networks and relationships [29]. Distinguishing between social, economic, and cultural capital, “[Bourdieu’s] conceptualization enriches the understanding of the body of commodities and resources that may be converted into what Sen terms *capabilities* …” [30] (p. 49).

### 1.2. Applying the CA to Assess Well-Being among Young Adults

Despite difficulties to operationalize the CA [21,34,35], several empirical studies on well-being based on the CA have been conducted among young adults. For instance, Bucheli [36] assessed multi-dimensional urban well-being among 18–28 year-olds in Bogota (N = 6998). The study included 14 dimensions that young people had considered as relevant for their well-being. Additionally, an overall capability index was constructed. Results revealed significant differences on the capability index between groups of 18–20-year-olds, 21–24-year-olds, and 25–28-year-olds. Age differences were also found for individual well-being dimensions, with younger participants facing higher difficulties in some domains (built environment, food security, inequality, and discrimination), with some of them partly diminishing with advancing adulthood (freedom and independence, and leadership and participation). Additionally, the author reported a significant influence of the quality of place (socio-economic strata) on how capabilities were exercised. Multivariate regression analysis including further variables to predict the overall capability index confirmed the positive association between better socioeconomic strata and a higher capability score. Significant effects were also found for ethnicity, dominance of a second language, and place of birth. Compared to the youngest respondents, only the age group of 21–24-year-olds showed a significant positive effect in the regression model. The study did not investigate associations between financial aspects and capabilities. In another study [37], the author assessed socio-spatial differences in young adult’s well-being on the basis of a spatialized index of capabilities. Results showed that capability scores were lower in neighborhoods characterized by lower educational achievements. Additionally, being male and living in areas with higher socio-economic strata enhanced capability scores. Finally, Chen and Lin [38] examined well-being dynamics among poor 8–24-year-olds in Taiwan (N = 8278) over time on the basis of capability indicators and functionings. The authors differentiated between secured well-being states, capability deprivation (deprivation of opportunities), functioning deprivation (inability to meet basic needs), and double deprivation (deprivation of capabilities and functionings). Deprivation indexes were calculated for individual capability and functioning dimensions. Additionally, deprivation indexes of dimensions were aggregated into indexes of capability and functioning deprivation. Although not focusing on young adulthood, the results of the multivariate analysis showed that the likelihood of capability deprivation decreased with increasing age. Conversely, younger respondents were more likely to exit functioning or double deprivation than older respondents. Moreover, the provision of pocket money, the possession of a personal computer, social relationships, and a higher educational level of parents seemed to have protective effects against any form of capability, functioning, or double deprivation. 

### 1.3. Measuring Capabilities and Functionings

Depending on the availability of data, capabilities have often been assessed using functionings or proxy measures for capabilities for practical reasons [34,35]. Anand and van Hees [39] developed a questionnaire to directly assess both self-reported valuation of one’s capabilities and one’s satisfaction with related functionings in seven life domains. The authors [39] examined the distribution of capabilities and related achievements among an older British adult population. Among the study sample, participants were most satisfied with the capabilities of health and environment. Interestingly, results from multivariate analysis showed that satisfaction with overall capabilities decreased with higher income. In multivariate analysis of individual functionings, only ethnicity showed a significant effect on one functioning, e.g., the functioning of health. Further, associations between capabilities and related functionings all were significant. Anand and van Hees’ questionnaire was also applied by Van Ootegem and Verhofstadt [40], who aimed at investigating the potential of self-reported capabilities for the study of well-being among N = 483 18-year-old students in Belgium. Congruent with the CA theory, the findings showed that the majority of the capabilities were larger than their related functionings. Exceptions were found for the areas of “happiness” and “personal integrity”, wherein the relationship was inverse, with functionings being greater than capabilities. According to the authors, these functionings might possibly be not the result of choice made out of a set of larger capabilities. Regression analysis on overall capabilities revealed significant effects of parental factors (strictness and marital status), extraversion, and mood of the day. The authors concluded that subjective capabilities are measurable and that empirical distinctions can made between self-reported capabilities and functionings. To analyze how self-reported capabilities and functionings are perceived to differ, Al-Janabi [41] modified the original version of the ICECAP-Capability measurement tool to assess both capability and functionings for five attributes of a person’s life among a United Kingdom-based convenience sample. Results showed that capabilities were larger-than functionings, equaled functionings, or were smaller-than functionings. Moreover, education and health affected the reporting of perceived differences between capabilities and functionings. Capabilities finally varied more with education, while functionings varied more with health status.

Drawing from a large sample of young adult men, this study analyzed their multi-dimensional well-being, spanning an evaluative space of self-reported capabilities and functionings across different life domains. Using repeated cross-sectional data from the Young Adult Survey Switzerland (YASS) allowed us to investigate well-being and its correlates among a large and representative group of young men over time. The survey encompasses male Swiss citizens of conscription age (19–25 years), from rural and urban areas, and from almost all socio-economic groups. It includes persons fit for military service, persons unfit for military service, and persons who opted for civilian alternative service. The sample comprises healthy and unhealthy participants and further allows for a more detailed analysis of population sub-groups such as, for example, according to age. The results based on these monitoring data collected between 2010 and 2015 might provide useful insights into the associations between socio-economic and demographic factors and CA-based well-being among 18–25-year-olds over time. 

In analyzing the data, our assumptions were the following: First, the study describes distributions of capabilities and related functionings according to individuals’ capital equipment. On the basis of the literature [37,38,41], we assumed that higher educational assets (cultural capital) might enhance capabilities in the respective life domains. As indicated by Chen and Lin [38], this association might also be observable between cultural capital and functionings. Although research findings on the association between financial means (economic capital) and capabilities and functionings are not unanimous [38,39], we assumed that economic capital will enhance both measures, as indicated by research on young men’s wellbeing [13]. To our knowledge, there is thus far no study investigating associations between social capital, capabilities, and functionings. Referring to the importance of social support and relationships for young people [13,38], we assume that more social capital positively affects both capabilities and functionings. Additionally, this study was interested in the question if capabilities are consistently larger than the corresponding functionings, as suggested by CA theory. On the basis of earlier studies [40,41], we assumed that in certain domains of life, capabilities and functionings might show patterns that are contrary to CA theory.

Second, and to verify the theoretical assumption of capabilities and functionings being empirically different constructs [40] and non-perfectly correlated due to the element of choice [26], we assumed correlation coefficients between capabilities and functionings to be smaller than 1.0.

Third, multivariate linear regression analysis was conducted to verify the associations of capabilities and functionings with the different types of capital, taking into account further co-variates known to influence young men’s well-being. On the basis of earlier studies [36,37,38], we assumed that the observed bi-variate associations might also be observable in the multivariate model. While the influence of age on the perception of capabilities and functionings has been reported in multivariate models [37,38], the age group of 18–20-year-olds was mostly considered in aggregated form. This study investigated if differences in capabilities or functionings can also be found between groups of 18-, 19-, and 20+-year-olds. We assumed that perceived capabilities and functionings might increase with increasing age, as suggested by previous study results [37,38]. Additionally, differences between rural and urban residence will be analyzed as place of residence has been proven to influence well-being [36,37]. The multi-variate models were finally controlled for survey year in order to identify potential changes in capabilities and functionings over time. We used overall capabilities and overall functionings as dependent variables in the multivariate models. The overall capability item used in this study has been shown to represent a satisfactory aggregate measure of the seven singular capability domains among the present study population [42]. Further, and as shown above, aggregate measures of functionings (deprivation) were used to assess correlates of multidimensional well-being among young people.

## 2. Method

Data for this study were obtained from the male sample of the cross-sectional Young Adults Survey Switzerland (YASS) conducted in 2010, 2011, 2014, and 2015 [43,44]. The survey aims at monitoring changes in attitudes and values with regard to education, work, leisure time, politics, well-being, and life perspectives among young adults. As young women’s participation in military service is voluntary, the number of women in the sample is small, and we decided to use only the male participants’ data. We focused on respondents from the three major language regions (Italian, French, and German). The general aims, concepts, and design of the survey have been described in more detail elsewhere [45,46].

### 2.1. Participants

Across all study waves, a total of N = 64,608 (2010 N = 17,262; 2011 N = 14,162; 2014 N = 23,048; 2015 N = 10,136) male respondents participated in the survey. The results were based on male respondents who completed all capability and functioning items (N = 58,615). Respondents who did not provide answers to at least one of the items in the capability and functioning item batteries were excluded from the analysis (N = 5993, 9.27%). We analyzed the socio-demographic profiles of respondents and non-respondents to account for self-selection processes among survey participants. A higher percentage of non-respondents followed a lower secondary (14.98% vs. 8.93%) or vocational track (62.5% vs. 57.11%) compared to those who responded (see Table 1).

### 2.2. Procedure

The quadrennial YASS was administered through paper-and-pencil questionnaires and took place at six centers handling recruitment for the compulsory Swiss military service in all Swiss language regions (German, French, Italian, and Romansh). Additionally, a complementary sample representing about 5% of the female population of this age group was invited to take part in a postal survey. Both surveys were voluntary and anonymous.

### 2.3. Measures

#### 2.3.1. Capabilities and Functionings

To measure capabilities and functionings, YASS used Anand and van Hees’ [39] suggested set of items for capabilities and corresponding achieved functionings. Respondents were asked to self-report their valuation of capabilities in seven life domains—e.g., the perceived scope to seek happiness in life, to achieve things in life, to live a healthy life, to be adequately intellectually stimulated, to form satisfying social relationships, to live in pleasant environments, and to act with personal integrity—on a 7-point Likert scale ranging from 1 = very good to 7 = very inadequate. The capability instrument also included an item on the evaluation of one’s overall capabilities (“Taking all things together, I think my options are…”). In our analysis, and in line with Hofmann et al. [42], we used reverse coding to ensure an ascending order of responses (1 = very inadequate to 7 = very good; see Table 2). This capability instrument has been tested for psychometric properties in the context of YASS among N = 17,152 young men in three Swiss language regions [42]. Results confirmed a good internal reliability of the German, French, and Italian language versions, with Cronbach’s α = 0.853, 0.870, and 0.877, respectively. In all language versions, capability items showed correlations between 0.3 and 0.7 among each other, and all items were positively correlated with the overall capability item. Exploratory factor analysis revealed a single common factor solution, with all items demonstrating overlap with the identified factor and explaining between 53% and 58% of the variance in the different language regions. Strong correlations between the sum score of the seven capability items and the overall capability measure were found (0.651 for the German, 0.676 for the French, and 0.671 for the Italian version), indicating that both measures are closely associated. The variability in the seven capability domains predicted around 47% of the adjusted variability in the overall capability measure, indicating its aggregation of different capability domains in the YASS study sample.

In addition to the capability items, satisfaction with the seven corresponding functioning items was assessed. Respondents were asked to report their satisfaction with achievements in the seven life domains (i.e., “happiness”, “sense of achievement”, “health”, “intellectual stimulation”, “social relations”, “environment”, and “personal integrity”) on a 7-point Likert scale ranging from 1 = very good to 7 = very inadequate. Again, we used reverse coding (1 = very inadequate to 7 = very good; see Table 2). 

Lacking an overall-functioning item, an overall functioning index was constructed on the basis of all seven functioning items. The Cronbach’s alpha coefficient for the scale including seven functioning items was 86.8, and the item-to-total correlations were all above 0.5, ranging between 0.5457 and 0.6987, thus confirming satisfactory internal consistency [47]. Spearman rank correlations (r) between the seven functioning items were consistently positive and all greater than 0.3. They were therefore considered suitable to be included into a factor analysis. Correlations were highest (r = 0.664) between “achievement” (F2) and “happiness” (F1) and lowest (r = 0.363) between “social relations” (F5) and “health” (F3). Most items showed moderate associations between r = 0.4 and r = 0.6 (see Table 3 for the correlation matrix). Moreover, a Kaiser–Meyer–Olkin measure of sampling adequacy (KMO) of 0.894 (>0.6) and a significant Bartlett’s test of sphericity with Ch2 (21; 20782) = 62,584.353 and *p* < 0.001 confirmed the suitability for factor analysis. Following Hofmann et al. (2013) and Krishnakumar and Nagar [48] (p. 490), we then conducted an exploratory factor analysis using principal axis factoring as the simplest latent variable model. We selected the number of factors based on eigenvalues greater than 1. We found a single common factor solution. All seven domain-specific items were loaded between 0.5 and 0.7 on this single factor. “Happiness” showed the highest (0.756) and “health” (0.585) the lowest loading onto the single factor. Communality estimates for each domain ranged between 0.4 and 0.6. On the basis of these results, we constructed a functioning sum score based on the mean values of the seven domain-specific functioning items. The score ranged from 1 to 7 with a mean of 6.02 (SD = 0.87). Overall capabilities and the functioning sum score showed a moderate correlation (r = 0.6, *p* < 0.001). 

#### 2.3.2. Social Capital

Social capital was measured by the number of persons from which the participant could borrow CHF 500 within two days. The number of persons, including parents, partners, siblings, grandparents, friends, and colleagues, who could be asked to lend money was transformed into a sum score ranging from 0–15 persons.

#### 2.3.3. Economic Capital

As most respondents were still in education and not yet fully engaged in working life, we used the parents’ financial situation as an indicator of economic capital. Respondents’ perceived financial situation of their parents was categorized into 1 = “humble”, 2 = “moderate”, 3 = “decent”, and 4 = “very good”.

#### 2.3.4. Cultural Capital

The young adults’ own *academic track* was measured by the current or expected level of post-mandatory education according to the International Standard Classification of Education (ISCED) scale from 2A to 5A [49]. It was categorized into 1 = “lower secondary”, 2 = “vocational training”, 3 = “grammar school”, and 4 = “tertiary training”.

All analyses were controlled for age (categorized into 1 = “18 years old”, 2 = “19 years old”, and 3 = “20+ years old”), survey year (1 = “2010”, 2 = “2011”, 3 = “2014”, and 4 = “2015”), and place of residence (1 = “urban”, 2 = “rural”).

### 2.4. Statistical Analysis

Descriptive statistics for the distribution of the eight self-reported capability items and their related functioning items were displayed according to individuals’ equipment with social, economic, and cultural capital. Next, the strength of bivariate correlations between capabilities and functionings were assessed. As both variables were measured on an ordinal scale, we used the Spearman correlation test, which additionally does not require any specific distribution of the data for the analyses. Finally, we ran linear regression analyses to study the multivariate associations between the three independent forms of capital and the dependent overall capability measure (model I). We used the overall capability item (“Taken all things together, I think my options are…”), which has been shown to be a suitable aggregate measure of different capability domains [40,42,50], as the dependent variable. In regression model II, we assessed the multivariate correlations between a dependent overall functioning measure and the three independent capital measures. As the YASS questionnaire does not include an overall measure of functionings, we constructed an index score based on the mean values of the seven individual functioning items (F1–F7). Before constructing the index, we computed Cronbach’s alpha coefficient to assess the internal reliability for the seven functioning items. Spearman rank correlations between all functioning items were assessed, and exploratory factor analysis was conducted to assess their underlying factor structure. All regression models were adjusted for age, residence, and survey year, as described above. Analyses were conducted using STATA version 16.1 [51].

## 3. Results

### 3.1. Descriptive Statistics

#### 3.1.1. Distribution of Capabilities and Functionings According to Cultural Capital (Own Academic Track)

**Figure 1:**
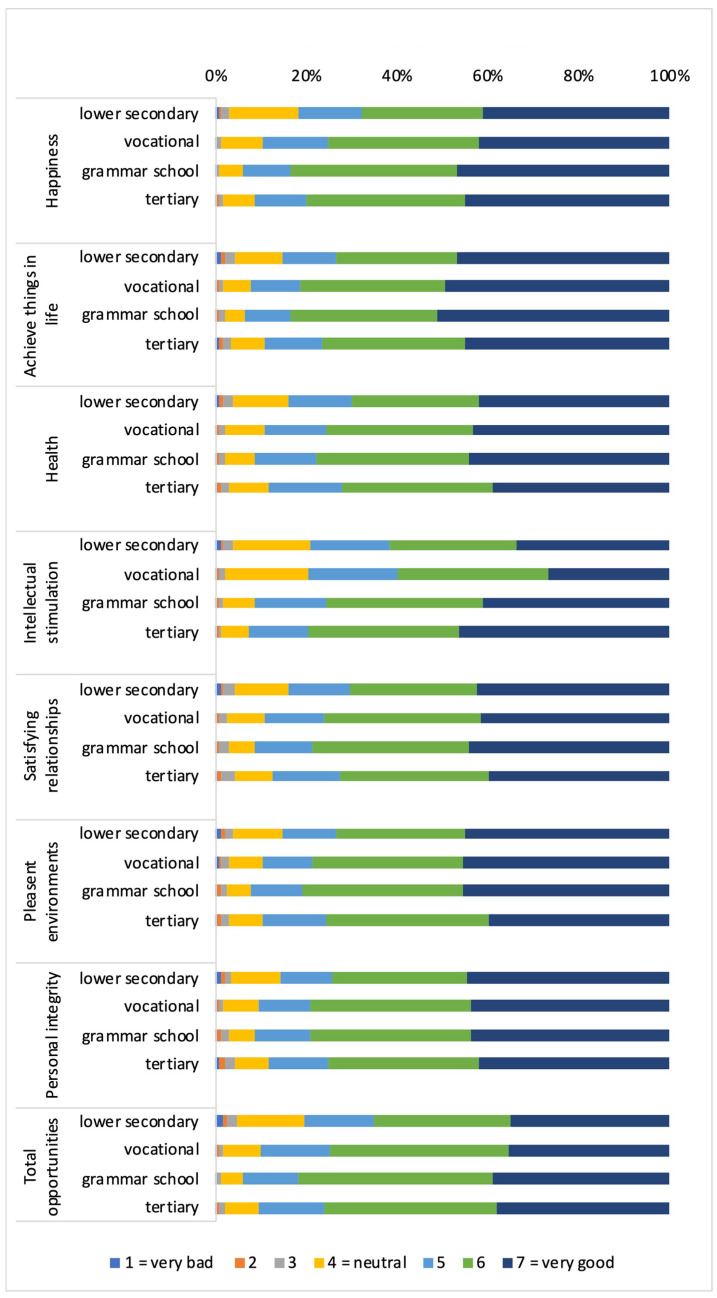

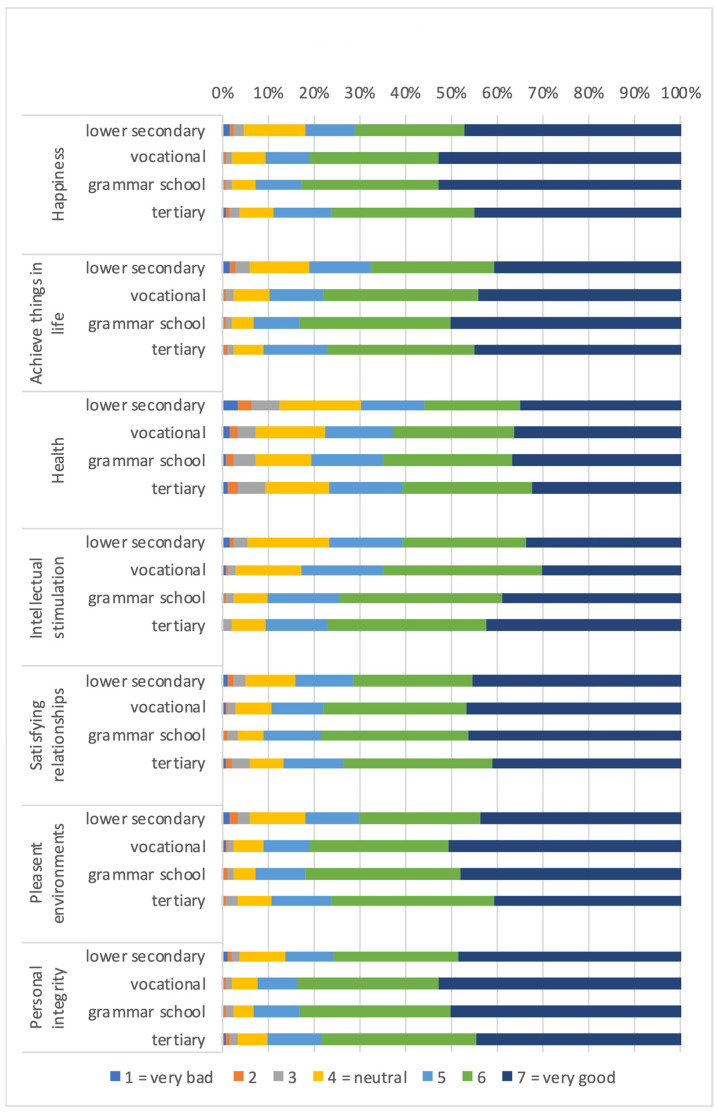
Distribution of capabilities and functionings according to cultural capital (own academic track). (**a**): Distribution of capabilities; (**b**): Distribution of functings.

As Figure 1 shows, the majority of respondents rated their capabilities and related functionings as good or very good. Results showed a u-shaped distribution of capabilities, with higher percentages of those attending grammar school or vocational training rating their capabilities as good or very good compared to their counterparts who followed a lower secondary or tertiary track. An exception is the capability for intellectual stimulation. Its perception improved linearly with educational level. Moreover, the percentage of persons who perceived their overall capabilities as good or very good increased with educational level.

With regard to the corresponding functionings, the results showed comparable u-shaped patterns except for the functioning of intellectual stimulation. The functioning to be healthy was perceived less often as good or very good than the corresponding capability across all educational levels. An inverse observation was made for the area of personal integrity: more persons rated their functioning to act with personal integrity as good or very good when compared to the related capability. The functioning to be happy was more often considered as good or very good in comparison with the corresponding capability only by those in secondary education or in vocational training.

#### 3.1.2. Distribution of Capabilities and Functionings According to Economic Capital (Parental Financial Situation)

**Figure 2:**
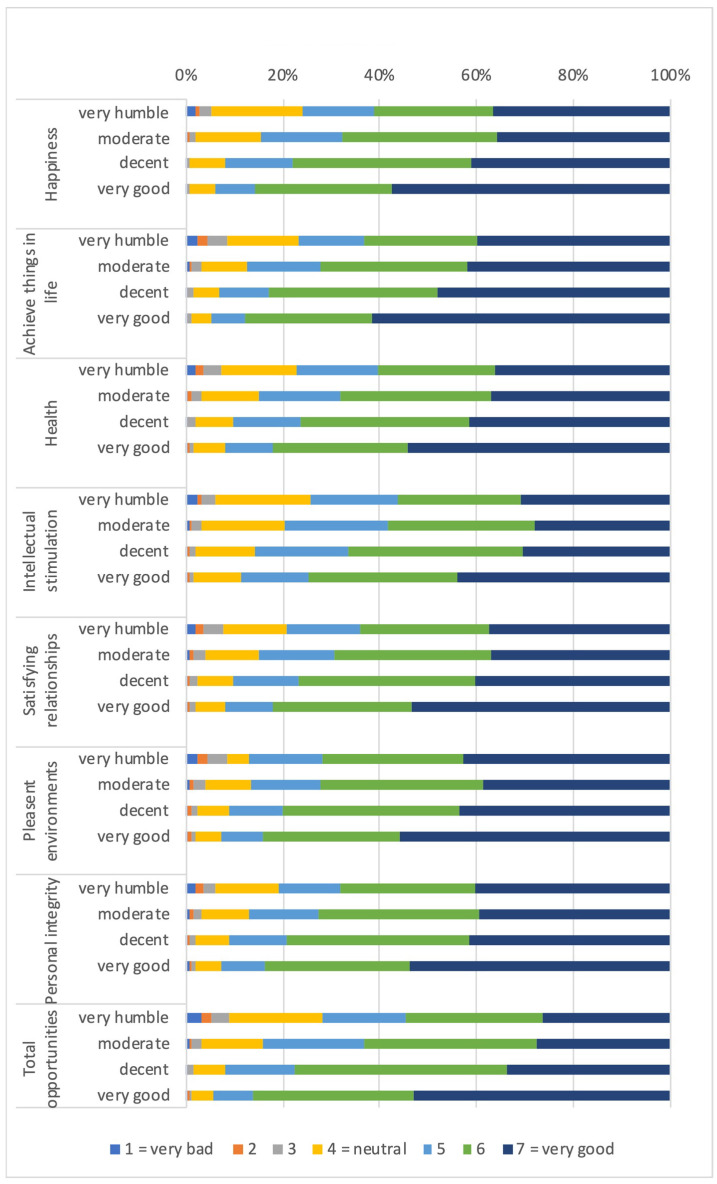

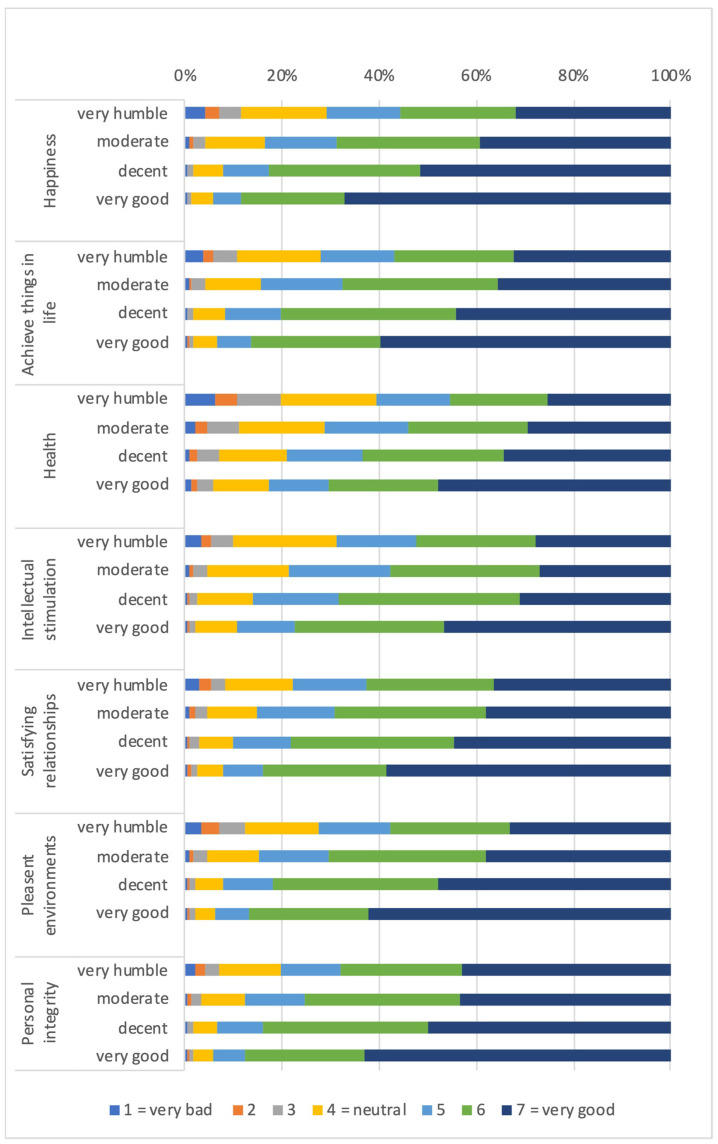
Distribution of capabilities and functionings according to economic capital (parental financial condition). (**a**): Distribution of capabilities; (**b**): Distribution of functings.

Capabilities and functionings in seven life domains all linearly increased with improving financial situation (see Figure 2). While the capability to achieve things in life was considered as good or very good by the majority of those in a very good financial situation (87.71%), the majority of those in a very humble financial situation (68.15%) considered the capability to act with personal integrity as good or very good. The perception of overall capabilities improved linearly with better perceived parental financial situation. Across all financial conditions, the functioning to be healthy was less often considered as good or very good in comparison with the corresponding capability.

#### 3.1.3. Distribution of Capabilities and Functionings According to Social Capital (Number of Persons to Borrow 500 CHF From)

**Figure 3:**
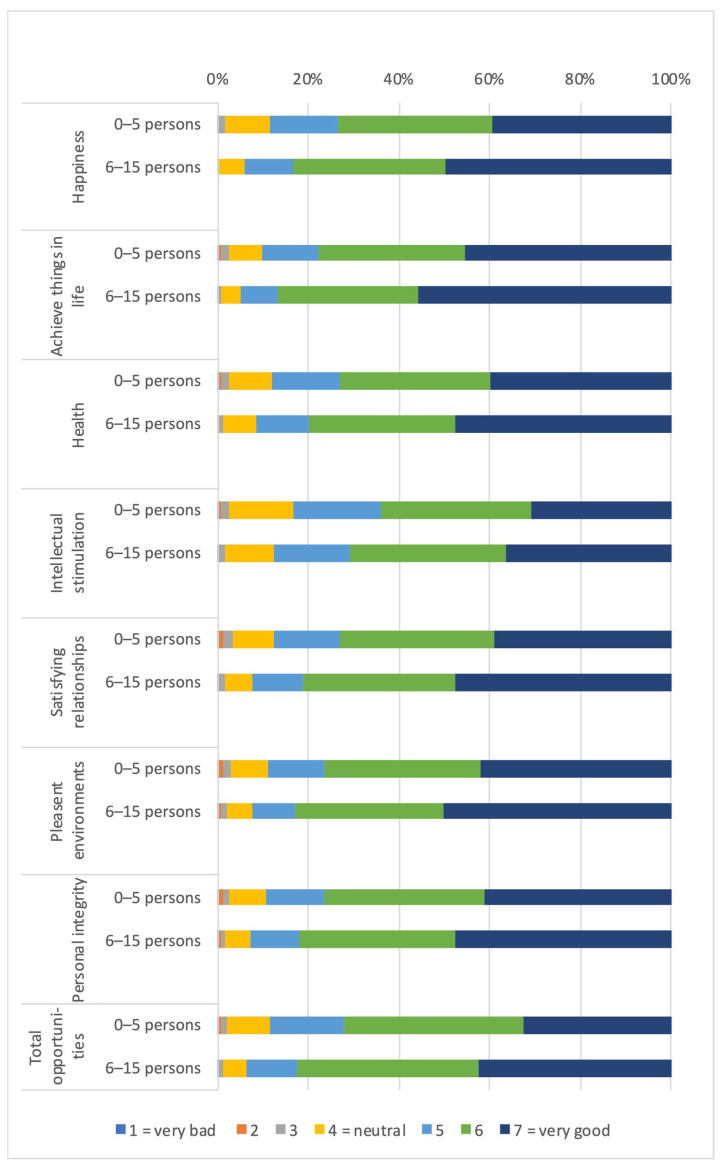

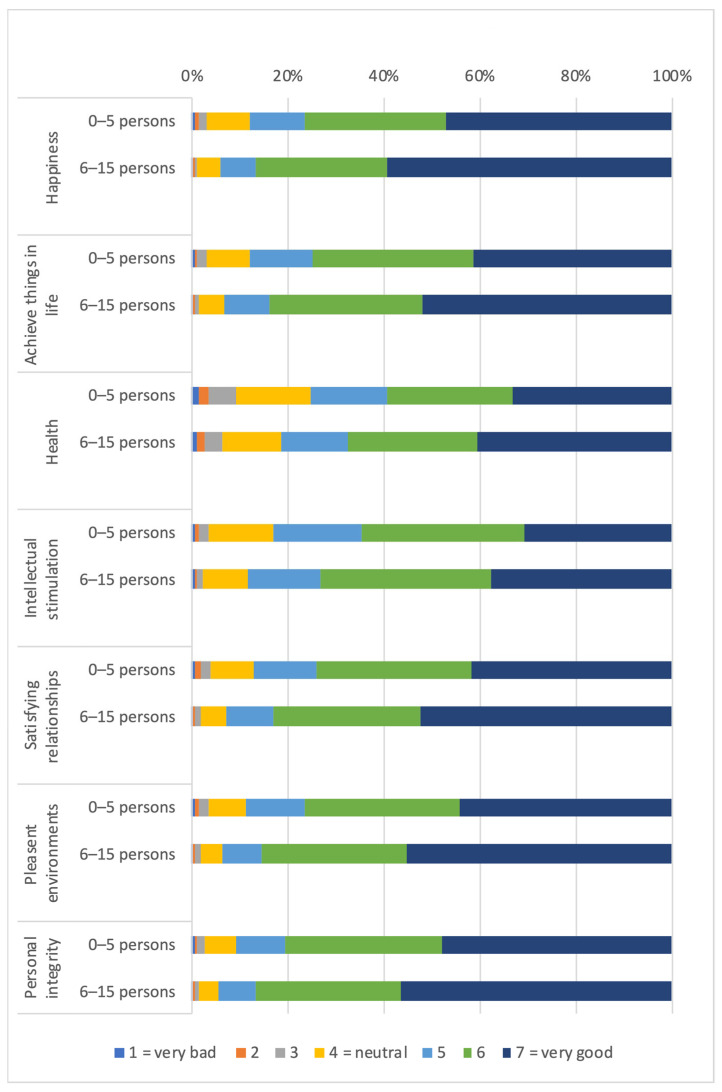
Distribution of capabilities and functionings according to number of persons to borrow CHF 500 from (social capital). (**a**): Distribution of capabilities; (**b**): Distribution of functings.

Among those reporting to know 6–25 persons to borrow money from, higher percentages reported that their capabilities and functionings in seven life domains were good or very good when compared to their counterparts (see Figure 3). While 72.22% of those with 0–5 persons to borrow money from considered their overall capabilities as good or very good, 82.26% of their counterparts did so. In both groups, the functionings to be happy and to act with personal were perceived more often as good or very good when compared to the corresponding capabilities. An inverse pattern was found to the area of health: the functioning to be healthy was less frequently considered as good or very good compared to its corresponding capability.

### 3.2. Correlations between Capabilities and Functionings

Spearman rank correlations (r) between the seven capability and the corresponding functioning items were all of moderate strength and ranged between r = 0.43 (for the capability to be happy and the related functioning of being happy) and r = 0.57 (for the capability to form satisfying relationships and the related functioning of having satisfying relationships). Overall capabilities and overall functionings also showed a moderate association (r = 0.60). A zero-order correlation matrix including all capabilities and functionings is presented in Table 3.

### 3.3. Multivariate Analysis of Social, Economic, and Cultural Capital on Overall Capabilities (Model I) and Overall Functionings (Model II)

Table 4 presents the adjusted associations between the three types of capital and overall capabilities (model 1), as well as overall functionings (model 2). The regression coefficients showed similar patterns in both models. Following vocational training, grammar school or a tertiary education track (as compared to following a lower secondary track) were all positively associated with a significant increase of capabilities in model 1, and of functionings in model 2. Significant increases on both dimensions were also found for increasing economic capital and increasing social capital. Being 19 or 20+ years old was associated with significantly lower capabilities and functionings when compared to being 18-year-olds. A significant increase in capabilities and functionings was found among persons living in rural areas compared to those in living in urban environments. Finally, capabilities and functionings tended to increase significantly since 2010.

With regard to the respective importance of the included variables, standardized ß-coefficients revealed that economic capital showed the strongest positive association with both overall capabilities (ß = 0.3127, *p* < 0.001 for a very good and ß = 0.2513, *p* < 0.001 for a moderate financial condition) and functionings (ß = 0.3405, *p* < 0.001 for a very good and ß = 0.3011, *p* < 0.001 for a decent financial condition). In model 1, economic capital was followed by grammar school (ß = 0.1382, *p* < 0.00), social capital (ß = 0.1168, *p* < 0.001), and a moderate financial condition (ß = 0.0926, *p* < 0.001). In model 2, economic capital was followed by social capital (ß= 0.1415, *p* < 0.001), a moderate financial condition (ß = 0.1278, *p* < 0.01), and grammar school (ß = 0.1214, *p* < 0.001).

## 4. Discussion

This study aimed at examining social correlates of young adult multi-dimensional well-being on the basis of Sen’s CA and Bourdieu’s theory of capitals. The first research question addressed the distribution of capabilities and functionings according to individual resources operationalized in terms of Bourdieu’s social, economic, and cultural capital. Generally, all survey respondents rated their capabilities and functionings in the different life domains as high. Our results showed respondents following a vocational training or grammar school track (cultural capital), perceiving their parents’ financial situation as good or very good (economic capital) and knowing 6–15 people to borrow money from (social capital) more often reported to consider their capabilities and functionings as good or very good. These findings support our assumption of the capability-enhancing effect of cultural and economic capital, which has also been found by other authors [13,37,38]. The u-shaped distribution of cultural capital found in our analysis might be explained by the fact that tertiary education often is a time characterized by financial restrictions and time-consuming study episodes, while following a vocational training is often financially reimbursed in Switzerland, providing young people with more financial freedom compared to non-paid student life. In addition, students in grammar school mostly still live at their parents’ home, which is associated with less financial worries and what might possibly influence the perception of actual capabilities and functionings. Social capital showed a positive correlation with both capabilities and functionings. This finding reflects the literature on the effects of perceived social resources and social networks on young people’s well-being [52,53].

The functioning to be healthy was rated lower than the corresponding capability to be healthy among all groups of social, economic, and cultural capital. However, and seemingly contradicting theoretical reasoning, reported capabilities were not always greater than their respective functionings in our study population. Across social and cultural capital levels, the functionings of being happy and to act with personal integrity were considered to be good or very good more often than their respective capabilities. No such differences were observed for economic capital. These observations are in line with Van Ootegem and Verhostadt [40], who also found higher functionings than capabilities in the domains of happiness and social integrity among young adults. While these results might appear partly “illogical” [41], Al-Janabi [41] also observed differences between capabilities and functionings varying with educational level. It is also possible that interaction mechanisms between capabilities and functionings are at work—capabilities and functionings might be a result or a prerequisite of one another, a theory we did not investigate in this study.

Second, and to gain a better understanding of the relationships between capabilities and their corresponding functionings among the study population, we assessed bi-variate correlations. Following Sen’s argument of individual freedom to choose a valuable functioning out of a set of capabilities [23], a capability and its related functioning might be correlated, but not in a perfect way. Our results showed moderate associations between each pair of related capability–functioning items, with correlations ranging from r = 0.43 for the “happiness” capability–functioning pair to r = 0.57 for the “satisfying relationships” capability–functioning pair. These findings are in line with Anand and van Hees [39], who reported a significant relationship between achievement satisfaction and its corresponding capability satisfaction. Additionally, we might assume that those capability–functioning pairs with the highest correlations might represent the most valued outcomes among our study population (e.g., satisfying relationships). This makes sense for our study population, as the quality of social relationships is highly supportive for young adult well-being and functioning [54]. Further, not only the relating capability–functioning pairs but also all the capability and functioning items were significantly correlated with each other. The response rates for self-reported capability and functioning items were quite high (>90%), which corresponds to the results of Al-Janabi [41]. The high response numbers suggest that Anand and van Hees’s [39] capability measurement tool might be easily manageable for young adults.

Finally, this study intended to explore multivariate associations between social, economic, and cultural capital and overall capabilities, as well as overall functionings. To assess overall-capabilities, we used the overall capability item (“Taken all things together, I think my options are…”) as the dependent variable, which has been shown to be a suitable aggregate measure of different capability domains [40,42,50]. With regard to the newly constructed overall functioning sum score, analysis of internal validity showed similar results as reported by Hofmann et al. [42] for the capability sum score. However, testing psychometric properties of the instrument was not the goal of this article and warrants further investigation. A correlation coefficient of r = 0.60 between overall capabilities and overall functionings suggests that both are closely correlated but still different proxies of a latent well-being variable. We did not consider perceived capabilities as measures of “real” capabilities. We followed Van Ootegem and Verhofstadt [50] in arguing that our study aimed at exploring possibilities to assess well-being in terms of self-reported capability and functioning aggregates rather than at investigating the extent to which capabilities represent actual opportunities.

Multivariate analyses confirmed the descriptive results but also revealed some differences between the models. In both models, all three forms of capital were significantly correlated with overall capabilities and functionings. The strongest association was found for economic capital in both models. Overall capabilities and functionings decreased with increasing age, which is contrary to other studies reporting an inverse pattern [36,37]. Capabilities and functionings were perceived as better by rural rather than urban residents. The role of place in shaping capabilities has been analyzed by Bucheli [36,37] and merits further research in the context of rural and urban environments. The results also indicated a tendency of significantly increasing capabilities and functionings over time. Given that young adults are often referred to as “carriers of social change” [36], it will be of great interest to explore in future studies if societal crises such as the current COVID-19 pandemic influence this pattern, resulting in differently perceived capabilities and functionings among this age group.

Models differed with regard to the relative importance of the capital variables—while social capital followed economic capital in importance in the overall functioning model, cultural capital was more important for overall capabilities. An explanation for this finding might be that information on perceived capabilities is more forward-looking while information on functioning is more backward-looking [50]. While money is needed for both perceived capabilities and functionings, cultural capital might enhance the perception capabilities in a future with a high educational degree. On the other hand, the feeling of being socially supported might enhance the satisfaction with functionings from a retro perspective.

### Limitations

The YASS data have several strengths, including the large number of available cases, their focus on young adulthood, and the inclusion of all social strata and major language regions in Switzerland. However, there are also several limitations. First, well-being was assessed using subjective measures of capabilities and functionings. Sen himself clearly advocates objectively measured functionings that are independent of individual evaluation [55] (p. 196). The challenge with subjectively measured functionings is that they may be rated as poor while they would be considered as moderate or even good from an objective perspective, or vice versa. This limitation is of special importance in our population group for which inconsistencies between the reporting of subjective well-being and the statistics of ill-health have been reported [15]. In this context, it would also be important to control for adaptation to restricted living conditions [56], personality traits, or mood of the day [50], which all have been reported to influence self-reports of capabilities and functionings. The self-reports possibly are also influenced by the usage of rather wide and unspecific capability and functioning items. For instance, Van Ootegem and Verhofstadt [40] included additional questions to the respective capability and functioning items to increase the questionnaire’s construct validity and concreteness. 

Further, we assumed that subjective capabilities are comparable between individuals, but opinions in the literature are divided regarding this issue (see, for example, [57]). Moreover, we did not operationalize “choice” in our model. Consequently, we cannot draw any conclusion at which occasion available capabilities have not been turned into functionings on the basis of individual choice or by other mechanisms such as adaptation to restricted living conditions. 

Another limitation is that the comparability of the overall capability and the overall functioning measure is not given. While the results of Hofmann et al. [42] showed that the aggregate capability measure captured the seven singular capability dimensions in a satisfactory way, we cannot draw a comparable conclusion for the assessment of functionings due to the lack of an overall functioning item in the questionnaire. Following the examples of Bucheli [36] and Anand and van Hees [39], future studies among young men should additionally examine individual capability and functioning dimensions to obtain a more detailed understanding of ongoing processes between well-being and social factors in different life domains.

Finally, this study is based on data collected between 2010 and 2015. Nevertheless, the congruency between this work’s findings and the empirical results of other studies investigating the associations between social correlates and CA-based well-being [36,38,39,40] suggests considering them as relevant also today.

## 5. Conclusions

Addressing the little researched population group of young adult men, this study aimed at contributing empirical evidence to the analysis of their well-being. Spanning a multi-dimensional evaluative space based on Sen’s CA, social correlates of well-being taking into account an individual’s social, economic, and cultural capital were investigated. Generally, young men in Switzerland rated their capabilities and functionings in the seven life domains as high. Moreover, the perceived capabilities and their correlating functionings were moderately correlated, as were measures of overall capabilities and overall functionings. These findings suggest that capabilities and functionings are similar albeit different concepts of well-being. Our findings empirically support the importance of the three forms of capital for both capabilities and functionings. Compared to their less privileged counterparts, respondents following a higher academic track, perceiving the parental financial situation as better than humble, and having a larger network to borrow money from more often reported their capabilities and functionings to be good or very good. The functioning to be healthy was considered lower than the corresponding capability to be healthy across all groups. However, capabilities were not always greater than their respective functionings (e.g., in the dimensions of happiness and personal integrity) and varied with cultural and social, but not economic, capital. Multivariate analysis confirmed the effect of social, economic, and cultural capital also for the perception of overall capabilities and overall functionings. Capabilities and functionings further decreased with increasing age, were higher among urban residents, and tended to increase over time. Minor differences between the models were found for the relative importance of the capital variables, which need to be further explored by future research among different study populations. In order for a better understanding of the mechanism at play in the transformation of capabilities into functionings to be developed, it would be of great interest to assess aspects of choice and of adaptation to restricted living conditions in future studies.

## Figures and Tables

**Table 1:** Characteristics of capability and functioning item-respondents and non-respondents.

Survey Respondents (N = 58,615)			Item-Non-Respondents (N = 5993)	
Own Academic Track	Frequency (%)	Mean (SD)	Frequency (%)	Mean (SD)
Lower secondary	5172 (8.93%)		863 (14.98)	
Vocational	33,077 (57.11%)		3601 (62.50)	
Grammar school	17,024 (29.39%)		1137 (19.73)	
Tertiary	2648 (4.57%)		161 (2.79)	
Missing	694 (1.18%)		231 (3.86)	
**Parental financial condition**				
Very humble	1868 (3.19)		228 (4.11)	
Moderate	10,583 (18.06)		1112 (20.06)	
Decent	32,723 (55.83)		2987 (53.90)	
Very good	12,181 (20.78)		1215 (21.92)	
Missing	1260 (2.15)		451 (7.52)	
**Number of persons to borrow money from**		5.72 (3.52), range 0–15		5.47 (3.77), range 0–18
Missing	3736 (6.37)		936 (15.62)	
**Age**				
18	16,514 (28.17)		1629 (27.18)	
19	24,785 (42.28)		2441 (40.73)	
20+	16,348 (27.89)		1778 (29.67)	
Missing	968 (1.65)		145 (2.42)	
**Residence**				
Urban	38,812 (66.22)		3955 (65.99)	
Rural	17,129 (29.22)		1706 (28.47)	
Missing	2674 (4.56)		332 (5.54)	
**Survey year**				
2010	15,752 (26.87)		1510 (25.20)	
2011	12,921 (22.04)		1241 (20.71)	
2014	20,810 (35.50)		2238 (37.34)	
2015	9132 (15.58)		1004 (16.75)	
Missing	-		-	

**Table 2:** Capability and functioning items.

*Capabilities—I Feel the Scope…*		1 Very Bad	2	3	4 Neutral	5	6	7 Very Good
1	to seek happiness in my life is							
2	to achieve things in my life is							
3	to live a healthy life, for my age, is							
4	for intellectual stimulation in my age is							
5	to form satisfying relationships in my life is							
6	to be in pleasant environments (taking home, work, and leisure together)							
7	to act with personal integrity in my life is							
***Functionings***								
1	Generally, my life is happy.							
2	I am satisfied with what I am able to achieve.							
3	I live a healthy life, for my age.							
4	I am adequately intellectually stimulated.							
5	I have satisfying social relations.							
6	I live, work, and undertake leisure activities in pleasant environments.							
7	I am able to behave in ways that do not comprise my personal integrity.							

**Table 3:** Spearman correlation matrix of capability (C) and functioning (F) items among and between each other (all correlations significant at *p* < 0.001).

Item		Functionings							Capabilities							
		F1 Happiness	F2 Achievement	F3 Health	F4 Intellectual stimulation	F5 Satisfying relationships	F6 Pleasant environments	F7 Personal integrity	C1 Happiness	C2 Achievement	C3 Health	C4 Intellectual stimulation	C5 Satisfying relationships	C6 Pleasant environments	C7 personal integrity	C overall
**Capabilities**	C1 Happiness	0.429	0.487	0.325	0.384	0.358	0.395	0.391	1.000							
	C2 Achievement	0.535	0.494	0.338	0.371	0.424	0.458	0.456	0.625	1.000						
	C3 Health	0.387	0.380	0.495	0.356	0.320	0.364	0.381	0.472	0.549	1.000					
	C4 Intellectual stimulation	0.310	0.350	0.289	0.548	0.305	0.304	0.313	0.447	0.434	0.463	1.000				
	C5 Satisfying relationships	0.409	0.386	0.270	0.374	0.571	0.422	0.410	0.459	0.538	0.431	0.466	1			
	C6 Pleasant environments	0.374	0.365	0.269	0.324	0.408	0.440	0.404	0.428	0.506	0.433	0.407	0.543	1		
	C7 personal integrity	0.363	0.374	0.290	0.340	0.381	0.405	0.491	0.426	0.482	0.422	0.385	0.485	0.550	1	
	C_overall	0.492	0.545	0.367	0.440	0.412	0.466	0.458	0.612	0.581	0.475	0.454	0.475	0.453	0.461	1
**Functionings**	F1 Happiness	1														
	F2 Achievement	0.664	1													
	F3 Health	0.443	0.455	1												
	F4 Intellectual stimulation	0.459	0.500	0.497	1											
	F5 Satisfying relationships	0.530	0.486	0.363	0.475	1										
	F6 Pleasant environments	0.564	0.532	0.400	0.446	0.576	1									
	F7 personal integrity	0.529	0.511	0.398	0.447	0.530	0.612	1								
	F_overall	0.787	0.781	0.703	0.730	0.745	0.775	0.729	0.523	0.579	0.516	0.459	0.535	0.486	0.497	0.600

**Table 4:** Multivariate regression analysis on overall capabilities (model1) and overall functionings (model 2).

	Model I		Model II	
	*Overall Capabilities*		*Overall Functionings*	
	Coeff. (se) ^a^	Stand. ß-Coeff.	Coeff. (se) ^a^	Stand. ß-Coeff.
**Own academic track**		-		-
Ref. = Lower secondary				
Vocational training	0.1924 (0.016) ***	0.0926	0.1838 (0.013) ***	0.1069
Grammar school	0.3099 (0.017) ***	0.1382	0.2254 (0.014) ***	0.1214
Tertiary training	0.2634 (0.026) ***	0.0546	0.1712 (0.021) ***	0.0429
**Financial condition parents**				
Ref. = Humble				
Moderate	0.2633 (0.027) ***	0.0990	0.2815 (0.022) **	0.1278
Decent	0.5234 (0.025) ***	0.2513	0.5190 (0.021) ***	0.3011
Very good	0.7894 (0.027) ***	0.3127	0.7117 (0.022) ***	0.3405
**Persons to borrow money from**	0.0344 (0.001) ***	0.1168	0.0345 (0.001) ***	0.1415
**Age**				
Ref. = 18 years				
19 years	−0.0574 (0.011) ***	−0.0276	−0.0548 (0.008) ***	−0.0138
20 years and older	−0.1191 (0.012) ***	−0.0521	−0.1445 (0.0167) ***	−0.0765
**Survey year**				
Ref. = 2010				
2011	0.0342 (0.012) **	0.0140	0.0120 (0.010)	0.0059
2014	0.0869 (0.011) ***	0.0397	0.0848 (0.009) ***	0.0468
2015	0.0476 (0.014) **	0.0164	0.0570 (0.012) ***	0.0237
**Residence**				
Ref. = Urban				
Rural	0.0234 (0.009) *	0.0105	0.0425 (0.008) ***	0.0230
Constant	5.082 (0.030) ***		5.163 (0.025) ***	
		N = 50,148; adj.R^2^ = 0.0711 F(13, 50,134) = 296.07, *p* < 0.001		N = 50,148; adj. R^2^ = 0.0842 F(13, 50,134) = 355.88, *p* < 0.001

Significance levels at *** *p* < 0.001, ** *p* < 0.01, * *p* < 0.05; ^a^ standard error.

## Data Availability

This publication is based on data collected by the research consortium as part of the YASS project of the Federal Youth Surveys ch-x (project management: Prof. Dr. Stephan Gerhard Huber, Institute for Education Management and Economics of Education at the University of Teacher Education Zug; research partner “Education, Work, and Profession”: PD Dr. Urs Moser, Institute for the Management and Economics of Education, Associated Institute of the University of Zurich; research partner “Health and Sport”: Prof. Dr. Dr. Thomas Abel, Institute for Social and Preventive Medicine at the University of Bern; research partner “Politics, Civic Education”: Prof. Dr. Sandro Cattacin, Département de Sociologie at the University of Geneva; see www.chx.ch; accessed on 10 December 2021). The data presented in this study are available upon request from the authors.
